# Effect of Load-Bearing Wall Material on Building Dynamic Properties

**DOI:** 10.3390/ma17246101

**Published:** 2024-12-13

**Authors:** Maciej Zajac, Krystyna Kuzniar, Tadeusz Tatara

**Affiliations:** 1 Institute of Technology, University of the National Education Commission, ul. Podchorazych 2, 30-084 Krakow, Poland; krystyna.kuzniar@uken.krakow.pl; 2 Faculty of Civil Engineering, Cracow University of Technology; ul. Warszawska 24, 31-155 Krakow, Poland; tadeusz.tatara@pk.edu.pl

**Keywords:** construction material, linear-elastic material, numerical analysis, finite element method (FEM), 3D model, dynamic properties, natural vibration frequency, natural mode shape

## Abstract

Nowadays, more and more buildings are being constructed from various types of modern materials. Many works have been written about these materials, which primarily focus on the influence of their properties on the thermal and acoustic insulation of, for example, building walls. However, there are very few publications analyzing the influence of construction materials on the dynamic properties of building structures and their vibration behavior. Yet, vibrations are dangerous for building structures. In the analysis of dynamic issues, the dynamic properties of objects should primarily be taken into account because the dynamic response of a building depends on the values of these parameters. This article focuses on numerically determining and analyzing the impact of load-bearing wall construction material on building dynamic properties—natural vibration frequencies and mode shapes. Seven building construction materials were considered, and then nine variants of building load-bearing walls made from these materials were analyzed. The analyses were carried out on the example of a low-rise administrative building structure. The building was modeled using the finite element method (FEM) with three-dimensional (3D) model analysis. Three variants of 3D FEM models were proposed, validated, and compared. A notable impact of load-bearing wall material properties on the natural frequencies and mode shapes of building structures was found. Two issues could be mentioned as the main new contributions of this paper: numerical analysis and comparing the effect of various building construction materials on dynamic building properties and the proposition and validation of various approaches to 3D FEM building load-bearing wall modeling. The findings of this research are of important significance and should be taken into account when constructing buildings subjected to dynamic loading or analyzing the possible harmful effects of various types of vibrations on buildings.

## 1. Introduction

Nowadays, more and more buildings are being constructed from various types of modern materials and using innovative construction solutions. There are many works being written about these materials. These works focus primarily on the influence of the properties of these materials on the thermal and acoustic insulation of, for example, building walls. However, there are very few publications analyzing the influence of the properties of construction materials on the dynamic properties of building structures and their vibration behavior. The lack of this type of paper highlights the need for further research. Meanwhile, in the surrounding, increasingly intensively developing world, the environment in which humans live is exposed to various negative impacts. These negative environmental influences include mechanical vibrations transmitted to structures and people in buildings who passively receive these vibrations.

Mechanical vibrations are dangerous for building structures and can negatively affect the human body of those who stay in buildings and passively receive these vibrations. In rapidly developing sustainable means of transport, reducing mechanical vibrations acting on buildings in the vicinity of communication routes (e.g., highways, metro lines, and railway lines) is a key task. For example, in the design of track surface structures (railway lines and metro lines), a system of block supports in a casing and vibration isolation mats is used. This solution provides, among other things, damping of vibrations caused by the passage of rail vehicles.

The design and use of buildings near railway lines, highways, metro lines, and tram lines also involve designing these objects in a way that limits vibration levels. These can be actions related to the design of surface structures with appropriately selected vibration isolation solutions or appropriate building design. Similar problems apply to buildings located in seismically active areas and mining regions.

In the analysis of dynamic issues, the dynamic properties of objects (natural vibration frequencies, corresponding mode shapes, and damping) should primarily be taken into account. The dynamic response of a building depends on the values of these parameters.

Natural vibration frequencies can be determined using several methods. These methods include analytical techniques, numerical approaches [1], measurement methods, and laboratory tests on shaking tables [2].

Numerical methods are essential for the dynamic analysis of buildings, particularly in determining natural vibration frequencies. The most widely used numerical tool is the finite element method (FEM), which divides the structure into smaller, simpler finite elements, making solutions easier to obtain. It is worth noting that the finite element method (FEM) is commonly used for analyzing various types of engineering problems. The FEM is utilized in mining for analyses related to determining the state of exploratory work for raw material deposits, as well as for computational determination of the stress–strain state of rock mass associated with the extraction process [3,4,5]. It is also used in the design work of installations in the chemical and energy industries, such as steam boilers used in power plants [6,7], and in fluid mechanics to solve, for example, gas flows [8]. The FEM is also applied to create a practical, convenient methodology, and in energy, the finite element method is used to calculate critical parts and components of rockets, aircraft, and dams, as well as to solve problems of filtration and thermal conductivity [9,10,11]. The FEM can also be used for sensitivity analysis of high-speed railway bridges. In FEM models of the track–bridge system, the ballastless track and trackless bridge model are considered [12]. Similar to [12], paper [13] is also concerned with the impact of earthquakes on structures, but it focuses on calculations of the proper distribution of the rolling friction coefficient. Analyses are conducted to improve the isolation performance of a spring–damper–rolling isolation system [13].

The Lanczos method [14], an iterative technique, is used to find the eigenfrequencies and mode shapes of large matrices. It is especially effective in modal analysis, where the objective is to ascertain the dynamic properties of the structure. A simplified method for estimating a fundamental natural frequency of a structure is the Rayleigh–Ritz method, which utilizes the distribution of mass and stiffness within the structure [15]. An advanced numerical technique for dynamic analysis is the Wilson method, enabling precise determination of natural frequencies and mode shapes while accounting for material and geometric nonlinearities [16]. The MAC (Modal Assurance Criterion) method is employed in modal analysis to evaluate the consistency between experimental and theoretical mode shapes. This tool is instrumental in verifying numerical analysis results against measurement data [17].

The natural vibration frequencies of buildings depend on numerous factors, including the building’s structure, the materials used in its construction, and ground conditions. The excitation of free vibrations (here equated with natural vibrations due to the low damping values in building structures) can be caused by various sources, such as road traffic, rail traffic, construction works, mining tremors, earthquakes, and even the use of the building. The natural vibration frequencies of a structure are significantly influenced by the type, density, and stiffness of the ground; the stratification of the ground substrate; the presence of groundwater [18]; and the interaction between the foundation and the ground [19]. The type of construction materials significantly affects the natural vibration frequencies of buildings, as the chosen materials can increase the stiffness, mass, and structural damping.

Especially, the behavior of building load-bearing walls substantially affects the dynamic properties of a whole building and the response of a structure subjected to dynamic loads. Load-bearing walls are the major resistance system of buildings located in wind- or earthquake-prone zones due to their high in-plane stiffness and strength. An important issue is the accurate location of walls. The size and placement of openings in shear walls significantly affect their behavior under dynamic loads as well.

The article focuses on numerically determining and analyzing the impact of load-bearing wall construction material on building dynamic properties—natural vibration frequencies and mode shapes. Seven building construction materials were taken into consideration, and then nine variants of building load-bearing walls were made from these materials. The analyses were carried out on the example of a low-rise administrative building structure. The building was modeled using the finite element method (FEM) and three-dimensional (3D) model analysis. Three variants of 3D FEM models were proposed and their results compared.

In particular, the new contributions of this paper could be summarized as follows: (1) numerical analysis and comparing the effect of various building construction materials on dynamic building properties; (2) proposition and validation of various approaches to 3D FEM building load-bearing wall modeling.

## 2. Materials and Methods

### 2.1. Applied Construction Materials

As mentioned above, buildings are constructed from various types of materials. The most commonly used material is concrete.

Concrete with additives is widely used in construction due to its improved mechanical properties and environmental aspects. An example of an additive used in concrete is CERTYD lightweight sintered aggregate, a porous ceramic aggregate produced using LSA (lightweight sintered aggregate) technology [20]. Lightweight concrete with the addition of CERTYD has a lower modulus of elasticity compared to normal-weight concrete with dolomite aggregate by about 62% after 90 days [20]. In practice, various types of concrete mixtures with recycled materials are used [21]. Research results indicate a significant impact of rubber granulation on the average compressive strength [21]. Adding 10% of 2/6 mm rubber granulate to the concrete mix is most beneficial in terms of strength, while adding 30% reduces the average compressive strength [21].

The strength modulus of concretes with additives depends on the type and amount of the additive (up to 5% of the cement mass). Concrete with fibrous additives can achieve a compressive modulus of elasticity in the range of 30–50 GPa, while concrete with microsilica additives can reach compressive and friction moduli of elasticity of 40–60 GPa and 1.5–2.5 GPa, respectively. Concrete with fibrous additives also improves the tensile modulus of elasticity, which can reach 10–20 GPa. The modulus of elasticity values for concrete with air-entraining and sealing additives range from 20 to 30 GPa and 25 to 35 GPa, respectively. The Poisson’s ratio also varies for different types of concretes with additives. The widest variability ranges are 0.20–0.25 for concretes with fibrous additives and 0.22–0.28 for those with air-entraining additives. Concretes with microsilica and sealing additives have Poisson’s ratio values in the range of 0.18–0.22 and 0.20–0.24, respectively [22,23]. High-performance concretes are increasingly used. In the study [24], a significant impact of the type of coarse aggregate on the deformation characteristics of high-performance concretes was found.

The literature also provides information on the impact of the type of coarse aggregate used in concrete on the value of the concrete’s modulus of elasticity [25]. This impact is a consequence of the multi-phase nature of concrete; the adhesion between the aggregate phase and the cement matrix is particularly significant, especially in high-strength concrete [24]. Modern concrete contains chemical additives constituting a small proportion (up to 5% of the cement mass) of the total mass. These additives significantly influence the parameters of the concrete mix (consistency and ability to fill formwork) and properties of hardened concrete (strength, durability, and frost resistance). For high-performance concretes, it is also important that they are made with high-strength aggregate and a high modulus of elasticity, which affects the value of the hardened composite’s modulus. A lesser-known relationship concerning the modulus of elasticity is the correlation between its value and the volumetric ratios of aggregate to hydrated cement paste in concrete of a given strength. Since the aggregate in such concrete has a higher modulus of elasticity than the hydrated cement phase, increasing the aggregate content in the concrete will raise its modulus of elasticity [26]. Temperature also affects the value of the concrete’s modulus of elasticity [27,28]. The modulus of elasticity remains constant in the temperature range of 21–96 °C; however, it significantly decreases at temperatures above 121 °C. This reaction may be due to the weakening of bonds within the concrete’s microstructure. The extent of the decrease in the modulus of elasticity due to temperature changes mainly depends on the type of aggregate used. Generally, the relationship between strength and modulus of elasticity of concrete and temperature is the same [26,27].

In the literature, there are also studies on determining the mechanical properties of high-strength lightweight concrete. In such concretes, polypropylene fibers are used as reinforcement. The reinforcement in these concretes is in the form of fibers from oil palm shells. The advantage of such concrete is the use of waste materials from palm oil production. The strength of such concrete is higher than that of traditional lightweight concrete [29]. The Young’s modulus for such concretes ranges from 12 to 15.4 GPa. This value is higher compared to concrete without any fibers [29].

Concretes with additives are widely used in transportation construction, such as road surfaces and railway tracks, where air-entraining additives enhance strength and durability. Concretes with sealing and air-entraining additives enable concreting at low temperatures and extreme conditions, while plasticizing additives and superplasticizers are used to produce high-quality concrete.

Due to the great popularity of concrete as a construction material, the first material analyzed in the article was standard reinforced concrete. The marking A was adopted for this material.

The high-strength oil palm shell lightweight reinforced concrete [29,30], marked as B, was taken into account as the next construction material considered. Using an oil palm shell in the lightweight concrete allows utilization of waste materials [29] and can make building structures more environmentally friendly. In addition, using such material reduces costs.

As the third type of analyzed construction materials, a cellular concrete [31,32] was proposed, and it was marked as C. This material, also called aerated concrete, porous concrete, and autoclaved aerated concrete, is very popular in building design of single- and multi-story objects because of its attractive mechanical properties (compressive strength), high thermal insulation, and soundproof insulation. Cellular concrete is produced using ordinary components: water, cement, gypsum, lime, and sand. An expanding agent (aluminum in a form of paste or powder) is also necessary in the standard production technology for foaming the cellular concrete.

A traditional and one of the oldest construction materials in masonry structures, i.e., brick, is also considered in the analysis. Notation D is applied for this case. Brick is a natural building material created in the process of burning a natural material, i.e., clay, with an admixture of refining substances. The popularity of brick is related to its high durability and universal application. It has a very high compressive strength. Moreover, it is also resistant to mechanical damage during transport and bricklaying. Brick is one of the materials least susceptible to shape changes, e.g., under the influence of static loads, heat, or low temperatures, and it provides electrical insulation. Brick is a non-flammable material. In addition, the use of traditional, natural raw materials and modern wall technology guarantees the well-being of the house’s inhabitants. The most modern production technologies also ensure a high level of environmental protection.

Moreover, three types of sand–lime bricks (calcium silicate materials) [33,34], marked using E, F, and G, respectively, were analyzed. Calcium silicate material is a building construction material that is a mixture made of natural ingredients: quicklime, quartz sand, and water. It stands out from other building structural materials primarily due to its low construction costs and the lack of harmful and radioactive compounds. There are some important advantages of using sand–lime bricks in building structures. One can mention, for example, good construction, thermal, fire resistant, and acoustic parameters. Thus, they are suitable for multi-story objects. In addition, they are among the most environmentally friendly building materials.

Therefore, seven different materials for the load-bearing walls of buildings were considered: standard reinforced concrete (A), high-strength oil palm shell lightweight reinforced concrete (B), cellular concrete (C), brick (D), sand–lime brick 1 (E), sand–lime brick 2 (F), and sand–lime brick 3 (G). The parameters of all analyzed construction materials are presented in Table 1.

In practice, the choice of construction material to be used in a given building depends not only on the type of building but also on, e.g., climatic conditions, the presence of groundwater, seismic activity, etc. Cellular concrete and sand–lime bricks are primarily used for constructing buildings designed to withstand static loads and wind gusts. These materials should not be used in environments exposed to groundwater or chemically contaminated water. Masonry buildings offer good thermal comfort; however, they are not suitable for construction in areas prone to seismic activity. Reinforced-concrete structures are commonly used for industrial constructions, such as hoist towers, dams, and buildings in seismic zones, due to their high resistance to both static and seismic loads.

### 2.2. Numerical Analysis

#### 2.2.1. Analyzed Variants of Building Load-Bearing Walls

**Figure 1:**
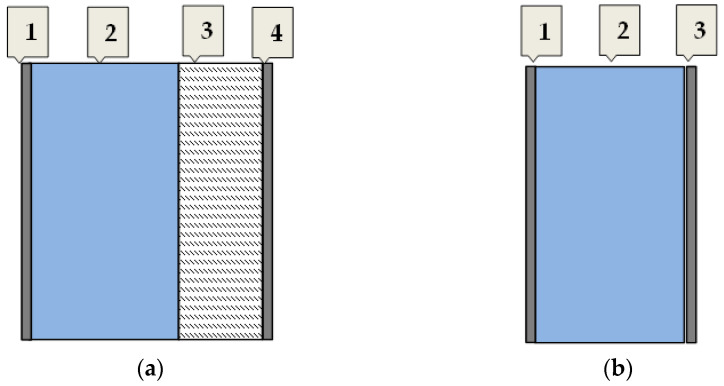
Pictorial diagram of layers of the analyzed load-bearing walls: (**a**) external (1, 4—cement–lime plaster; 2—load-bearing layer; 3—styrofoam); (**b**) internal (1, 3—cement–lime plaster; 2—load-bearing layer).

As was mentioned above, seven building construction materials for load-bearing walls were taken into consideration. In the case of all of the analyzed wall materials, each external wall has, in addition to the supporting (load-bearing) layer, a thermal insulation layer (styrofoam) and two layers of plaster, whereas all internal load-bearing walls consist of three layers only: the supporting (load-bearing) layer and two layers of plaster. Pictorial diagrams of layers of the analyzed external and internal load-bearing walls are shown in Figure 1.

Three different, commonly applied thicknesses of load-bearing layer were taken into account in the case of reinforced concrete (A) and one thickness for the other materials. Therefore, there are nine variants of building load-bearing walls from these materials. The thicknesses of the load-bearing layers for all considered wall variants and their designations are given in Table 2. Additionally, the thicknesses of the rest of the layers (thermal insulation layer and plaster layers) are also presented in Table 2.

The thicknesses of the load-bearing layers given in Table 2 are typical and very often used for the materials considered. However, not only for reinforced concrete but also depending on the type of building, other variants of these thicknesses are possible for all structural materials analyzed. For example, in the case of cellular concrete walls, load-bearing layers of 0.36 m thickness are also used; in the case of brick walls, load-bearing layers of 0.38 m thickness are also possible; and in the case of sand–lime bricks, load-bearing layers of 0.12 m, 0.22 m, and 0.25 m thickness are also practically available in buildings.

#### 2.2.2. Three-Dimensional FEM Building Model

**Figure 2:**
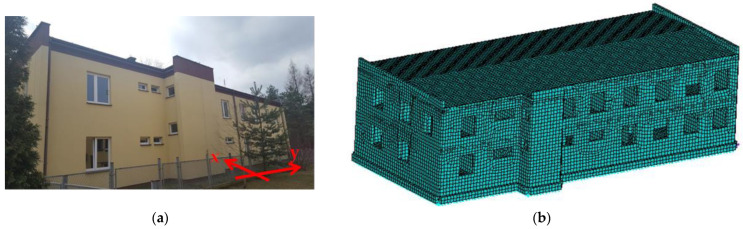
View of the analyzed building: (**a**) the actual object; (**b**) 3D FEM model.

A numerical analysis concerning the effect of load-bearing wall material on structure dynamic properties is based on a selected construction of the actual building structure. The view of this actual building is presented in Figure 2a. Additionally, in Figure 2a, the horizontal transverse (x) and longitudinal (y) axes of the building are marked in red.

The three-dimensional finite element method model (3D FEM model) of the building, including the applied finite element mesh, is shown in Figure 2b.

The finite element method software ANSYS [35] was used for the model creation. In this study, ANSYS software (v. 18.2) was applied to calculate the natural dynamic properties (frequencies and mode shapes) of a 3D building model. The interaction between the building and the ground was included by using spring elements with soil stiffness and damping. ANSYS was chosen because it offers many advanced options for dynamic modeling and allows the creation of parametric scripts. Using scripts made it faster to study the effects of different material properties for load-bearing walls on the natural vibration frequencies and mode shapes of buildings. This approach helped to save time and reduce mistakes that can occur when data are entered manually. The program also has powerful tools for calculating vibration frequencies and mode shapes while including damping, which allowed it to show how the building behaves under real conditions. The flexibility of ANSYS in defining material properties and boundary conditions, along with advanced dynamic analysis methods, was considered crucial for this study. The ability to add damping in soil spring models was especially important for simulating the interaction between the ground and the building, which is critical when studying the effects of mining tremors on structures.

A linear-elastic material behavior was assumed in the case of modeled elements of building structure. The relevant parameters of the analyzed load-bearing wall materials are summarized in Table 1.

To create the model of the building structure, two types of finite elements accessible in the ANSYS system were used: BEAM188 and SHELL181. The 2-node BEAM188 element with 6 degrees of freedom at each node was applied to model the continuous footings, foundation ties, and concrete ties, whereas the roof, ceilings, and walls (load-bearing walls and partition walls) were modeled using the 4-node SHELL181 element with 6 degrees of freedom at each node.

Three variants of models related to the considered load-bearing walls (see Section 2.2.1 were proposed, discussed, and analyzed. They were named as follows: Model 1, Model 2, and Model 3.

Model 1 takes into account only the stiffness of the load-bearing layer of the wall (the thickness of the wall is the thickness of the load-bearing layer), its mass, and the additional mass of non-load-bearing layers. For external walls, the mass of styrofoam and plaster is taken into account, and for internal load-bearing walls, the additional mass from plaster.

For Model 2, the average elastic modulus, Poisson’s ratio, and density for all wall materials were used. The wall thickness is the sum of the individual layer thicknesses (*di*). Thus, the equivalent value of elastic modulus, Poisson’s ratio, and density could be prepared according to Equation (1) [36]:

(1) $$Peqv=(\sum_{i}Pi\cdot di)/(\sum_{i}di),$$

where *Peqv* is the equivalent elastic modulus, Poisson’s ratio, or density, respectively; *Pi* is the elastic modulus, Poisson’s ratio, or density, respectively, of an individual layer; and *di* is the thickness of an individual layer.

In Model 3, each layer was included with assigned thicknesses and parameters reflecting the actual layer materials. Therefore, Model 3 is considered the most accurate of the three model variants.

In all models, the partition walls are made of brick, regardless of the material used for the load-bearing walls.

In the modeling of the subsoil, soil flexibility and damping were taken into account. Translation spring–damper elements COMBIN14 from ANSYS were applied for subsoil flexibility modeling in the horizontal (transverse and longitudinal) and vertical directions. The parameters (stiffness) for the spring elements were calculated on the basis of Savinov theory, using the Cz coefficient related to soil properties [37]. A value of dynamic soil parameter Cz = 50 MPa was assumed as a typical, frequently occurring characteristic of the building substrate. To determine the damping parameters, it was assumed that the soil density is 1800 kg/m^3^ and the shear wave velocity is 200 m/s. These parameters correspond well to the assumed value of the parameter Cz. In addition, the total foundation contact area was taken into account.

In this study, a 3D finite element model of the building was created using detailed architectural plans of an actual office building. The plans included ground floor and first-floor layouts, cross-sections of the foundations, as well as a vertical cross-section of the building. These documents provided the necessary information to accurately define the building’s geometry and structural elements.

The building is a two-story office structure without a basement, supported on reinforced-concrete strip foundations set at a depth of 1.4 m. The subsoil beneath the building consists of medium and fine sand layers, interspersed with yellow dust. The building’s dimensions in the plan are 12.7 m in the transverse direction (*x*-axis) and 29.9 m in the longitudinal direction (*y*-axis), with a total height of 7.3 m.

The structural system is based on a transverse–longitudinal layout of load-bearing walls, which ensure the stability of the building and are evenly distributed on both floors. The ground floor and first floor are further supported by slabs constructed of reinforced concrete.

The model includes all key parts of the building, starting from the foundations and moving up to the roof. Each component was assigned suitable material properties and finite element types to simulate realistic behavior under dynamic loads.

The foundations were modeled as reinforced-concrete strip footings using BEAM188 elements. These elements were chosen because they work well for simulating linear components like footings. Above the footings, the foundation walls were modeled as solid reinforced concrete using SHELL181 elements. These walls, with a thickness of 0.25 m, provide strong support between the ground and the rest of the building. The material used for the foundation walls had typical reinforced-concrete properties: an elastic modulus of 31.0 GPa, a density of 2500 kg/m^3^, and a Poisson’s ratio of 0.25.

The next part of the model is the ground floor. The load-bearing walls on this floor were also modeled with SHELL181 elements. To analyze how different materials affect the building’s vibration frequencies, several options for wall materials were included in the model. These options were standard reinforced concrete, brick, cellular concrete, and sand–lime bricks with various strengths. All partition walls, made of brick, were modeled using SHELL181 elements with a thickness of 0.12 m.

The first-floor slab was modeled as a reinforced-concrete plate using SHELL181 elements, with the thickness determined from the building design documentation. This slab was supported by load-bearing walls and ceiling ties. The ceiling ties were modeled using BEAM188 elements with a cross-section of 0.24 m by 0.24 m. These ties helped to evenly distribute loads and stabilize the structure. They were made of reinforced concrete with standard properties, including an elastic modulus of 27.0 GPa and a density of 2500 kg/m^3^.

The roof was modeled as prefabricated reinforced-concrete panels using SHELL181 elements. These panels were supported by load-bearing knee walls, completing the structural model.

The considered 3D FEM model was successfully validated using experimental data from in situ measurements in the case of a D-type load-bearing wall (traditional masonry, brick wall) as one of the analyzed material variants of load-bearing walls. The full-scale tests of the actual building (shown in Figure 2a) were performed in the Upper Silesian Coalfield (USC) region in Poland [38,39]. This region is characterized by very intensive seismic-type mining tremors. A detailed description of the model verification method is provided in [19].

## 3. Results and Discussion

The effect of load-bearing wall material on building dynamic properties was analyzed using calculated natural vibration frequencies and mode shapes. The focus was on horizontal vibration in two directions parallel to the horizontal transverse (x) and longitudinal (y) axes of the building (see Figure 2a) and torsional vibration of the building structure.

The natural frequency of horizontal transverse vibration, horizontal longitudinal vibration, and torsional vibration was denoted by fx, fy, and ft, respectively.

**Figure 3:**
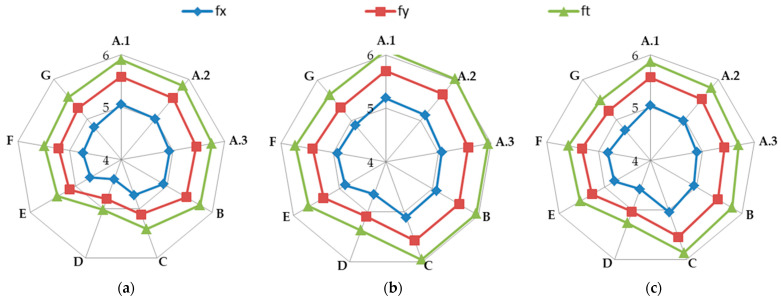
Building natural vibration frequency visualization in the case of the analyzed variants of building walls: (**a**) Model 1; (**b**) Model 2; (**c**) Model 3.

The calculated frequency results for Model 1, Model 2, and Model 3 are presented in Table 3. Additionally, these results are presented graphically in Figure 3.

A notable impact of load-bearing wall material properties on the natural frequencies of the building structure is visible.

In almost all calculation cases, regardless of the model used, the highest natural frequency values (fx, fy, and ft) were obtained for the load-bearing wall A.1 (standard reinforced-concrete wall with a thickness of 0.10 m). The only exception to the rule is the value of horizontal transverse frequency (fx) obtained for the load-bearing wall C (cellular concrete) using Model 3. In contrast, the lowest values (fx, fy, and ft) were calculated in the case of the load-bearing wall D (brick). This effect is visible for all applied models (see Table 3 and Figure 3).

The greatest influence of the material can be seen in the values of torsional vibration frequency (ft), regardless of the numerical model variant used. In the case of using Model 1, the highest value of ft calculated for reinforced-concrete wall A.1 differs from the lowest value of ft calculated for brick wall D by as much as 17.5%. Using the remaining two models gives slightly smaller differences—13.2% and 11.4% for Model 2 and Model 3, respectively.

The effect of construction material on the value of horizontal longitudinal vibration frequency (fy) is a little smaller. The differences in the highest and the lowest fy frequency values are 16.3%, 12.0%, and 10.7% for Model 1, Model 2, and Model 3, respectively.

The smallest influence of load-bearing wall material on natural frequencies was observed for the horizontal transverse frequency (fx). In this case, the differences in the highest and the lowest fx frequency values are 14.8%, 11.6%, and 10.3% for Model 1, Model 2, and Model 3, respectively.

At first glance, the differences of a few percent between the calculated natural frequency values for the adopted models may seem unrealistic. However, one must take into account the significant differences in the values of the modulus of elasticity and material density. This is particularly evident for the natural frequencies calculated for the material described as A, as well as C and D (see Table 1 and Table 3). The use of three load-bearing wall models does not result in significant differences either. Therefore, it is reasonable to adopt the simplified Model 1 or Model 2 in the analyses. Such an approach reduces the number of integration points in FEM elements used and shortens the calculation time. It should be noted, however, that in Model 3, the structural walls are described most accurately. However, from an engineering perspective, the natural frequency values of Model 1 or Model 2 can be accepted for analyses.

The impact of the analyzed material variants of building load-bearing walls on building natural mode shapes is shown in Table 4 using Model 1 as an example.

As you can see, the natural mode shapes of vibrations of the same building structure but made of different materials may differ significantly. A comparative analysis of the vibration modes calculated considering different construction materials listed in Table 4 indicates significant differences in these modes. This observation applies to horizontal transverse and longitudinal vibrations, as well as to torsion vibrations. In all these cases, there are clear differences in the color maps showing the displacements in mode shapes.

The analysis of the vibration modes corresponding to translational vibrations shows that in all cases, the largest relative resultant displacements occur in the extreme parts of the building in question. This information indicates where to expect the maximum dynamic response and potential damage to structural elements.

Similar natural mode shapes occur only in the case of building structures with load-bearing walls A.1, A.2, and A.3, i.e., standard reinforced-concrete walls, but of different thicknesses. For example, the differences in the highest displacements occurring in the case of A.1 and A.3 mode shapes are 4.5%, 5.7%, and 7.7% for transverse vibration (x), longitudinal vibration (y), and torsional vibration (t), respectively.

But in the case of a building structure with B-type load-bearing walls, these natural mode shapes are clearly different. Although the construction materials of A and B types are reinforced concretes, they differ in their properties (parameters), and this translates into differences in vibration forms.

Among all vibration modes in the group of horizontal transverse vibration modes, the group of horizontal longitudinal vibration modes, and also the group of torsional vibration modes, the natural mode shapes of the building structure with C-type load-bearing walls (cellular concrete) stand out clearly.

A detailed analysis of the vibration modes shows that the model made of cellular concrete C has the largest relative horizontal displacements. Conversely, the smallest relative displacements occur in the model made of reinforced concrete. For example, the differences in the highest displacements occurring in the case of C and A.3 mode shapes are 20.7%, 24.7%, and 16.8% for transverse vibration (x), longitudinal vibration (y), and torsional vibration (t), respectively.

Analyzing the vibration modes corresponding to torsional frequencies, we can state that these are complex vibrations. The largest calculated relative displacements occurred in the model made of cellular concrete C. This material should not be used in buildings where torsional vibrations may occur, for example, due to uneven dynamic loading.

The above observations regarding the influence of construction material on natural mode shapes formulated in the case of Model 1 also apply in the cases of using Model 2 and Model 3.

**Figure 4:**
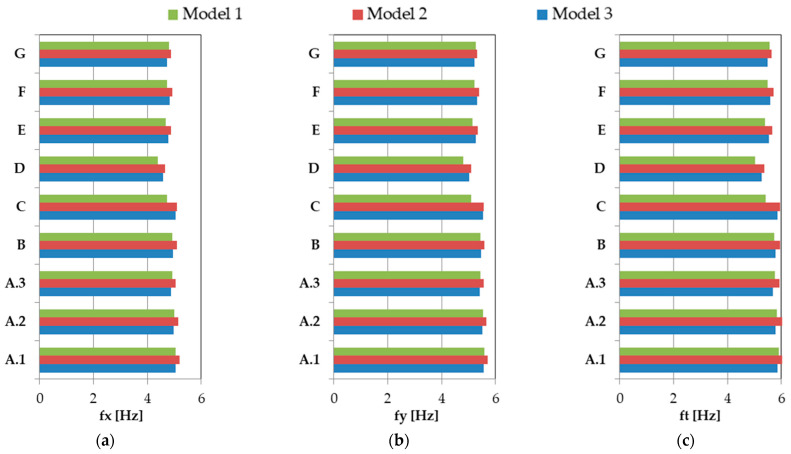
Comparison of natural vibration frequencies determined using Model 1, Model 2, and Model 3 in the case of the analyzed variants of building walls: (**a**) fx; (**b**) fy; (**c**) ft.

Additionally, results obtained using the three variants of 3D FEM models—Model 1, Model 2, and Model 3—were compared. Comparisons of the values of natural vibration frequencies determined in the case of the analyzed various building walls are presented in Figure 4.

Due to the fact that, among the three models considered, Model 3 most accurately represents the actual building structure, the natural frequency results obtained for the simplified Model 1 and Model 2 were compared with those from Model 3.

In general, the values of the natural frequencies fx, fy, and ft obtained using the three proposed models are very close. Thus, the average differences between the fx, fy, and ft frequency values calculated using Model 1 compared to Model 3 are 2.0%, 2.2%, and 2.4%, respectively. In the case of Model 2, these average, analogous differences in relation to the results from Model 3 are 2.5%, 1.7%, and 2.9% for fx, fy, and ft, respectively.

It is worth noting that in the case of both simplified models (Model 1 and Model 2), even the largest differences in the calculated frequencies in relation to the results for Model 3 are also relatively small. So, the ranges of these differences for the fx, fy, and ft frequencies are 0.2–6.5%, 0.2–7.8%, and 0.7–7.7% for Model 1, respectively. And for Model 2, these ranges are even smaller: 1.2–3.5%, 0.5–2.6%, and 1.5–4.4% in the case of fx, fy, and ft frequencies, respectively.

Furthermore, a comparison of building natural mode shapes determined using Model 1, Model 2, and Model 3 is shown for some exemplary representatives of the analyzed wall materials in Table 5 (for standard reinforced concrete A.1 load-bearing wall), Table 6 (for cellular concrete C load-bearing wall), and Table 7 (for brick D load-bearing wall).

A fairly good agreement between the corresponding vibration mode shapes determined using the three models can be observed. Small, only local differences are visible in the case of the natural mode shapes for Model 3.

## 4. Conclusions

This article presents the findings of natural frequencies calculated for applied variants of the administrative building model. The conclusions from the analysis are as follows:

- The main contribution of this study is the recognition of the influence of material parameters on natural lateral and torsional frequencies.
- The study highlights changes in natural frequency values which significantly affect a building’s dynamic response. The presented analysis results supplement research on the influence of different materials on natural frequencies of structures. The results are presented for a specific building with bearing walls. A notable impact of load-bearing wall material properties on the natural frequencies of building structures was observed. The greatest influence of the material can be seen in the values of torsional vibration frequency (ft). The effect of construction material on the value of horizontal longitudinal vibration frequency (fy) is slightly smaller. The smallest influence of load-bearing wall material on natural frequencies was observed for the horizontal transverse frequency (fx).
- The natural mode shapes of vibrations of the same building structure but made of different materials may differ significantly. This observation applies to horizontal transverse and longitudinal vibrations, as well as to torsion vibrations. Relatively similar natural mode shapes occur only in the case of building structures with standard reinforced-concrete load-bearing walls of different thicknesses. The natural mode shapes of the building structure with cellular concrete material of load-bearing walls stand out clearly regarding lateral and also torsional vibrations.
- Three models of the building using the FEM were adopted. The first model considers only the stiffness of the load-bearing layer of the wall; the second model consists of the average elastic modulus, Poisson’s ratio, and density for all wall materials; and the third includes each layer with assigned thicknesses and parameters reflecting the actual layer materials.
- The results of the calculations have led to significant conclusions. These findings should be taken into account when constructing new buildings subjected to dynamic loading. The structural walls in Model 3 are described most accurately. However, from an engineering perspective, the natural frequency values of Models 1 and 2 can be accepted for further dynamic analyses.
- In general, the values of all natural frequencies obtained using the three proposed models are very close. The average differences between the frequency values calculated using Model 3 compared to simplified Model 1 and Model 2 do not exceed a few percent. A fairly good agreement between the corresponding vibration mode shapes determined using the three models can also be observed.
- The analysis results indicate small differences in the values of natural vibration frequencies. This is due to changes in E simultaneously with significant changes in material density.
- The authors intend to include the following activities in further research on the topic under consideration: extension of the scope of analyzed materials, structures and the thicknesses of load-bearing walls; similar studies on other types of buildings, e.g., high-rise and medium-rise buildings; preparation of mathematical models that allow for the calculation and forecasting of the considered output parameters during the regression analysis; and study of the possibilities of using artificial neural networks for solving the considered problem.

## Figures and Tables

**Table 1:** Parameters of analyzed construction materials.

Material	Material View	Material Notation	Elastic Modulus [GPa]	Density [kg/m^3^]	Poisson’s Ratio [-]
Standard reinforced concrete	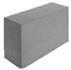	A	31.0	2500	0.25
High-strength oil palm shell lightweight reinforced concrete	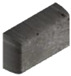	B	13.4	1900	0.20
Cellular concrete	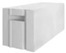	C	1.80	600	0.25
Brick	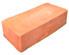	D	2.85	1800	0.25
Sand–lime brick 1 (class 20)	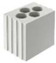	E	5.56	1820	0.23
Sand–lime brick 2 (class 15)	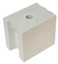	F	6.68	1810	0.21
Sand–lime brick 3 (class 15)	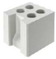	G	6.94	1730	0.23

**Table 2:** Thicknesses of wall material layers in the case of the analyzed building walls.

Construction Material	Analyzed Load-Bearing Wall	Layer Thickness [m]						
		External Wall				Internal Wall		
		1	2	3	4	1	2	3
A	A.1	0.015	0.10	0.15	0.02	0.015	0.10	0.015
	A.2	0.015	0.12	0.15	0.02	0.015	0.12	0.015
	A.3	0.015	0.15	0.15	0.02	0.015	0.15	0.015
B	B	0.015	0.15	0.15	0.02	0.015	0.15	0.015
C	C	0.015	0.25	0.15	0.02	0.015	0.25	0.015
D	D	0.015	0.25	0.15	0.02	0.015	0.25	0.015
E	E	0.015	0.18	0.15	0.02	0.015	0.18	0.015
F	F	0.015	0.18	0.15	0.02	0.015	0.18	0.015
G	G	0.015	0.24	0.15	0.02	0.015	0.24	0.015

**Table 3:** Building natural vibration frequency in the case of the analyzed variants of building walls.

Analyzed Load-Bearing Wall	Natural Vibration Frequency [Hz]								
	Model 1			Model 2			Model 3		
	fx	fy	ft	fx	fy	ft	fx	fy	ft
A.1	5.05	5.58	5.90	5.19	5.70	6.08	5.04	5.57	5.86
A.2	5.01	5.53	5.83	5.14	5.65	6.02	4.98	5.51	5.79
A.3	4.93	5.45	5.75	5.06	5.56	5.94	4.89	5.42	5.69
B	4.93	5.43	5.73	5.09	5.58	5.95	4.95	5.47	5.78
C	4.72	5.11	5.41	5.11	5.57	5.95	5.05	5.54	5.86
D	4.40	4.80	5.02	4.65	5.09	5.37	4.58	5.03	5.26
E	4.68	5.14	5.40	4.88	5.35	5.67	4.79	5.28	5.54
F	4.74	5.21	5.48	4.92	5.39	5.72	4.82	5.32	5.58
G	4.80	5.28	5.56	4.89	5.32	5.64	4.74	5.23	5.48

**Table 4:** Building natural mode shapes in the case of the analyzed variants of building walls (Model 1).

Analyzed Load-Bearing Wall	Natural Mode Shape		
	Horizontal Transverse (x) Vibration	Horizontal Longitudinal (y) Vibration	Torsional (t) Vibration
A.1	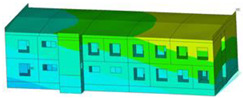	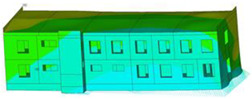	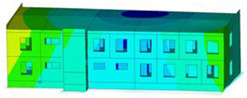
A.2	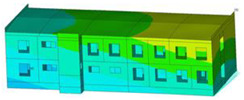	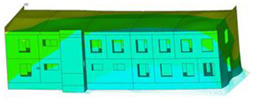	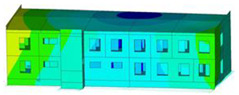
A.3	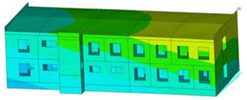	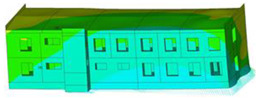	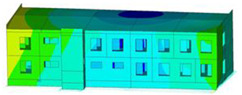
B	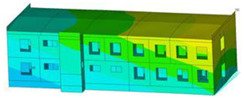	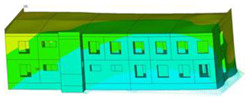	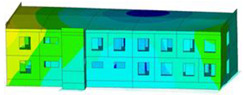
C	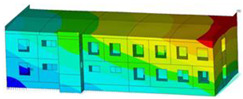	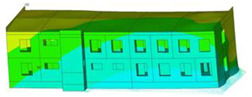	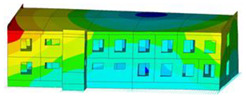
D	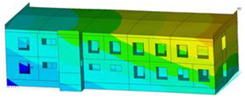	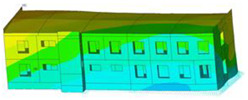	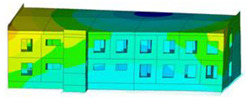
E	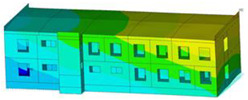	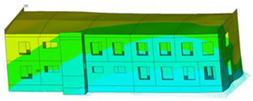	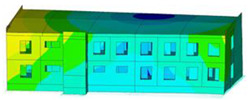
F	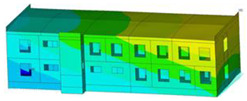	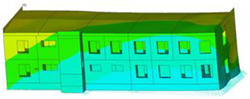	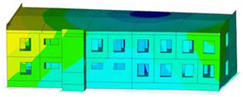
G	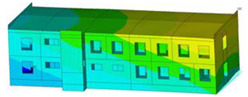	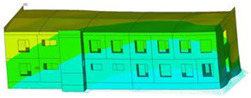	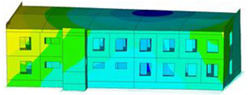

**Table 5:** Comparison of building natural mode shapes determined using Model 1, Model 2, and Model 3 in the case of the building structure with the A.1 (standard reinforced concrete) variant of load-bearing walls.

Model	Natural Mode Shape		
	Horizontal Transverse (x) Vibration	Horizontal Longitudinal (y) Vibration	Torsional (t) Vibration
Model 1	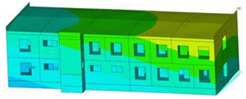	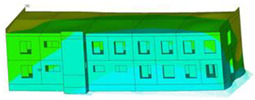	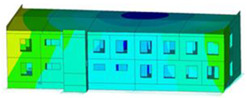
Model 2	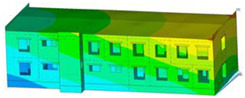	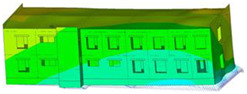	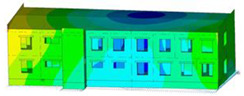
Model 3	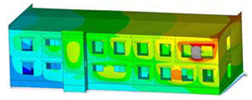	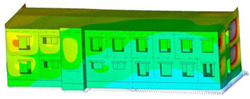	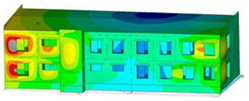

**Table 6:** Comparison of building natural mode shapes determined using Model 1, Model 2, and Model 3 in the case of the building structure with the C (cellular concrete) variant of load-bearing walls.

Model	Natural Mode Shape		
	Horizontal Transverse (x) Vibration	Horizontal Longitudinal (y) Vibration	Torsional (t) Vibration
Model 1	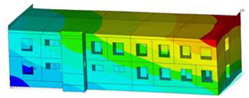	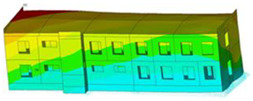	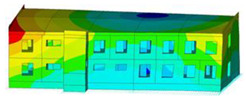
Model 2	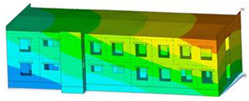	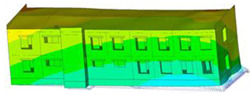	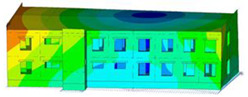
Model 3	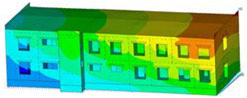	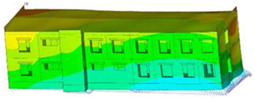	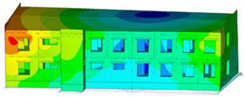

**Table 7:** Comparison of building natural mode shapes determined using Model 1, Model 2, and Model 3 in the case of the building structure with the D (brick) variant of load-bearing walls.

Model	Natural Mode Shape		
	Horizontal Transverse (x) Vibration	Horizontal Longitudinal (y) Vibration	Torsional (t) Vibration
Model 1	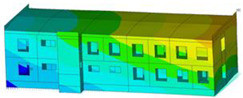	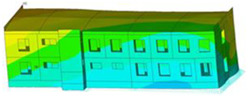	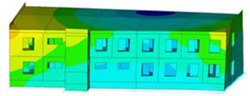
Model 2	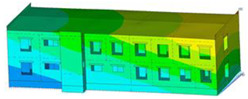	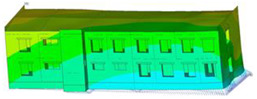	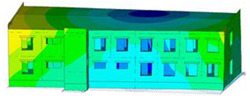
Model 3	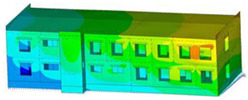	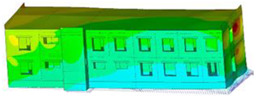	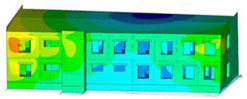

## Data Availability

The original contributions presented in this study are included in the article. Further inquiries can be directed to the corresponding author.
